# Human Posture Transition-Time Detection Based upon Inertial Measurement Unit and Long Short-Term Memory Neural Networks

**DOI:** 10.3390/biomimetics8060471

**Published:** 2023-10-02

**Authors:** Chun-Ting Kuo, Jun-Ji Lin, Kuo-Kuang Jen, Wei-Li Hsu, Fu-Cheng Wang, Tsu-Chin Tsao, Jia-Yush Yen

**Affiliations:** 1 Department of Mechanical Engineering, National Taiwan University, Taipei 106319, Taiwan; 2 Missile and Rocket Research Division, National Chung Shan Institute of Science and Technology, Taoyuan 325204, Taiwan; 3 School and Graduate Institute of Physical Therapy, National Taiwan University, Taipei 106319, Taiwan; 4 Mechanical and Aerospace Engineering, Samueli School of Engineering, UCLA, Los Angeles, CA 90095, USA; 5 Department of Mechanical Engineering, National Taiwan University of Science and Technology, Taipei 106319, Taiwan

**Keywords:** human posture change detection, deep learning, feedforward neural network (FNN), long short-term memory (LSTM), inertial measurement unit (IMU), internal sensing, human activity recognition (HAR)

## Abstract

As human–robot interaction becomes more prevalent in industrial and clinical settings, detecting changes in human posture has become increasingly crucial. While recognizing human actions has been extensively studied, the transition between different postures or movements has been largely overlooked. This study explores using two deep-learning methods, the linear Feedforward Neural Network (FNN) and Long Short-Term Memory (LSTM), to detect changes in human posture among three different movements: standing, walking, and sitting. To explore the possibility of rapid posture-change detection upon human intention, the authors introduced transition stages as distinct features for the identification. During the experiment, the subject wore an inertial measurement unit (IMU) on their right leg to measure joint parameters. The measurement data were used to train the two machine learning networks, and their performances were tested. This study also examined the effect of the sampling rates on the LSTM network. The results indicate that both methods achieved high detection accuracies. Still, the LSTM model outperformed the FNN in terms of speed and accuracy, achieving 91% and 95% accuracy for data sampled at 25 Hz and 100 Hz, respectively. Additionally, the network trained for one test subject was able to detect posture changes in other subjects, demonstrating the feasibility of personalized or generalized deep learning models for detecting human intentions. The accuracies for posture transition time and identification at a sampling rate of 100 Hz were 0.17 s and 94.44%, respectively. In summary, this study achieved some good outcomes and laid a crucial foundation for the engineering application of digital twins, exoskeletons, and human intention control.

## 1. Introduction

Currently, with the rapid development of smart-life technology, the trend of smart sensing has shifted from the field of external sensing (such as smart-home appliances) to the field of the internal sensing of the human body [1]. Human posture-change detection (HPCD) is an essential aspect of human–robot interaction research, involving collecting and analyzing signals generated by human body movements [2]. Precise and rapid prediction of human intentions can greatly enhance coordination between humans and machines, enabling collaborative robots, digital twins in the industrial field, diagnostic systems, rehabilitation devices, and even everyday-life assistance with greater ease and efficiency [3,4,5].

In addition to human–robot interaction, identifying human gait or posture is crucial for controlling exoskeletons. Exoskeletons typically employ two levels of control to ensure safe operation. The lower-level control utilizes feedback-based impedance or admittance control to assist joint movements [6]. However, during the process, even a small movement in the pilot’s joint may require a significant torque force from the actuator. To avoid using high feedback gain, one often selects the feedforward control technique. However, using a high feedback gain can lead to instability and result in pilot injury. Feedforward control requires prior knowledge of the motion trajectory; therefore, many commercial exoskeletons must rely on the pilot to switch between operating modes.

The focus of this study is on the application of lower-limb exoskeleton control. Optimizing posture-change detection is especially important, because if there is a delay between the exoskeleton control action and the user’s intention, the delay could cause sluggish movement and even injury to the pilot [7,8]. Therefore, it is crucial to develop methods for sensing the user’s intentions and detecting posture changes in real time to ensure safe and effective use of the exoskeleton. This future research aims to address these issues and improve the autonomous execution of exoskeleton systems.

To correctly detect human intention, there are two issues to address. The first issue is having adequate means to measure body movements. In recent decades, many researchers and commercial devices for external or internal sensing of human signals have been available, with different ways to collect body signals. Commercial external sensing systems like optical flow [9], edge contour [10], motion-history image [11,12], spatio-temporal volume [13], and the human alternative model [14] are image-based methods, and a fixed location for data gathering is required. On the other hand, some systems, such as the inertial-measurement-unit (IMU) based method [15,16,17], the electromyography (EMG) based method [18], and the flexible-sensor-based method, have some advantages regarding their ability to be wearable and to allow subjects to move around while gathering data. From the survey results for the hardware environment, it might be evident and appropriate to choose wearable-form tools to achieve the goals of human posture-change detection from the exoskeleton control system [19,20]. Recently, some research has focused on information from various sensors, such as the above-mentioned IMU, EMG, electrogoniometer (EGM), and so on, which have been integrated for effective action recognition. However, these also require data accumulation and analysis through moving windows and other methods [21,22,23]. Also, compared with other types of wearable sensing tools, the data captured by IMU is related to angle changes of the human torso, which is a more beneficial characteristic as it is not limited by individual differences of users, and its computable amount would be lower in the gait or posture-detection research field [7,15,19,20].

The second issue regarding human posture-change detection is the posture identification algorithm. A suitable algorithm that provides fast recognition and good judging accuracy is desirable; however, the highly nonlinear nature of the problem, in addition to the stringent time constraint, made it very difficult to solve with conventional methods. People have used machine-learning or statistical methods to attain the best identification results. Machine learning methods include K-nearest neighbors, mean shift, decision tree, Bayes classifier, and support vector machine (SVM) [24,25]. Statistical methods with advanced knowledge analysis of kinematics and kinesiology include the self-similarity matrix method and hidden Markov model. This research employed some of the most popular approaches, which are also used in gait detection or human activity recognition (HAR) [26,27,28]. Additionally, there has also been much research using neural network (NN) techniques such as convolutional neural networks (CNNs), recurrent neural networks (RNNs), and deep learning [15,29,30,31].

Archetti et al. and Ragni et al. compared performances between linear discriminant analysis (LDA) and random forest (RF) in predicting the intended reaching of the target with subjects wearing electromagnetic sensors [32,33]. Li et al. used action recognition, action prediction, and posture-change detection to predict the pitcher’s choice of one of the nine-square divisions by capturing and analyzing the pitcher’s RGB image and optical flow [34]. These results mainly concern the movements of the upper limbs. However, these results have focused on identifying the movements rather than obtaining a more precise time to predict intentions utilizing artificial intelligence (AI) or statistical tools. What is more, for timeseries-type data with specific postures and particular features, these methods might not consider the time-delay risk in implementation, and the machine learning or statistic methods above might not be suitable.

The authors of this research used the Long Short-Term Memory (LSTM) method to address these problems. LSTM is a recurrent neural network designed to process and predict events with long intervals and delays. It has a unique structure that enables it to perform better than general recurrent neural networks, especially in tasks such as autonomous speech recognition [35]. LSTM is not limited to speech-recognition tasks; it can also be applied in other areas, such as predicting human decisions [36], exoskeleton control, and posture-change detection for other equipment [37,38,39]. When the human body performs a posture change (whether it is wearing an exoskeleton or there is human–computer interaction), there is a linkage and correlation between each of the joints of lower limbs, and there is also continuity between and memory of various movements. The deep learning method of LSTM has the memory characteristics of recurrent neural networks, and can also avoid gradient-disappearance and gradient-explosion problems during long-sequence or long-time training. The characteristics of this study also focus on the determination of both specific continuous posture-transition time and status while other machine learning literature in the same field involve posture-status detection only. What is more, the accuracy of action recognition is relatively inconsistent in the existing literature [40]; so, the LSTM network structure is more suitable, and it is also the main reason and motivation for choosing the LSTM structure in this study.

This study conducted by the authors focused on the use of LSTM in exoskeleton control and posture-change detection. To evaluate the efficiency of LSTM in comparison to other algorithms, the authors also compared its performance with the linear feedforward neural network (FNN) method, a widely applied algorithm in AI research.

The primary goal of this research was to develop an algorithm that could detect changes in human postures with high precision and in a very short time. The ultimate aim was to reduce the time it takes for the algorithm to identify a new movement, enabling the exoskeleton control system to quickly switch to the new gait or posture, hoping to avoid causing danger, such as falling, or a situation in which, for example, the lower limbs are raised at an inappropriate moment or similar due to a misjudged strategy by the controller. This study also needs to consider the time delay for software and hardware integration. In addition, another focus of this research is how to achieve this goal with the smallest variety of sensors. This research used IMU sensors as the main measurement tool for several reasons. Firstly, these sensors provide a non-invasive way of detecting changes in posture, and they can be easily integrated with existing exoskeletons. Secondly, IMU sensors offer high data rates and good measurement resolutions, which are crucial for the fast detection of posture changes. Interestingly, the research team found that very few studies have focused on using only IMU sensors for the detection of the precise time of posture changes, despite these sensors being commonly used as wearable gait sensors to distinguish different human actions. In some cases, IMU sensors were used in conjunction with other sensor types to improve detection accuracy [17,19,20,40,41,42].

By developing an algorithm that can quickly and automatically recognize new postures, the pilot could enjoy a more comfortable ride without noticing the switches between different operating modes. It is worth noting that the response time of the algorithm was defined as the total time taken from when the recognition label was established by the algorithm to when the amount of data was input to the algorithm.

This research involved conducting experiments with IMU sensors attached to the right leg of subjects to analyze the changes among three different movements: standing, walking, and sitting. The researchers measured the joint angular displacements, angular velocities, and angular accelerations to evaluate the performance of the algorithm in detecting changes in human posture. The research team also compared the performance of two deep learning methods, FNN and LSTM, to assess which method could more accurately detect changes in posture and shorten the response time of the algorithm. The ultimate goal of this research was to contribute to the development of real-time human posture-change detection and exoskeleton control using simple sensor types such as IMU sensors.

In summary, this research is distinguished by its attributes and substantial contributions:

- It stands out in its precise identification of specific postures, relying solely on IMU data to capture the intricate dynamics within the human body, obviating the need for external sensors;
- A central focus of this study revolves around the meticulous prediction of transitions between different postures. This pioneering innovation holds the promise of facilitating seamless and secure transitions, whether in the context of exoskeleton utilization or the synchronization of digital twins in future applications;
- The development of a tailor-made deep learning framework designed for the detection and prediction of human intentions opens vast possibilities in the domains of digital twins, exoskeleton technology, and human intention control.

## 2. Materials and Methods

### 2.1. The Experimental Structure

The main objective of this study was to develop a systematic method for accurately detecting changes in human intentions and predicting the transition time using inertial measurement units (IMUs) and artificial intelligence technology. To achieve this objective, the researchers divided the experimental design into three parts.

The first part involved designing a protocol for clinical trials with IMUs. This protocol would specify the number and placement of IMUs on the human body to collect data on the movements and changes in posture. The second part of this study focused on data preparation and treatment to build a better model using artificial intelligence technology. The researchers collected data from the IMUs and used various techniques to preprocess the data, such as filtering, smoothing, and normalization. They then used machine learning algorithms to build a predictive model to accurately detect changes in human intentions. In the third part of this study, the researchers used the deep learning model to predict the transition time of human intentions. This involved analyzing the data collected from the IMUs to detect changes in posture or movements and predicting the time it would take for a human to transition from one intention to another.

### 2.2. The IMU Equipment

**Figure 1:**
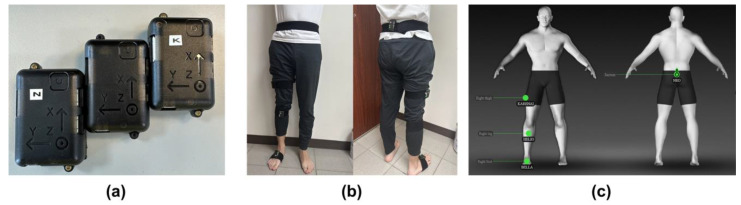
(**a**) The IMU equipment: NGIMU; (**b**) the front and back view of the subject wearing IMU sensors; (**c**) the setting of IMU sensors displayed in the iSen 3.0 analysis system.

The IMU equipment used in this study is NGIMU from x-io Technologies, Bristol, UK. Each NGIMU contains a 3-axial gyroscope, 3-axial accelerometer, and 3-axial magnetometer, as shown in Figure 1a. The sensor’s static roll, pitch, and yaw accuracy are less than 2°, and the maximum sampling rate is 400 Hz [43,44,45]. The system provided instant sagittal, transverse, and coronal plane angles when our subject wore the sensors on his right leg. The NGIMU would also be used with the iSen 3.0 analysis system from the STT system company, San Sebastián, Spain.

### 2.3. Participants

A convenience sample of 30 healthy subjects was recruited from July 2021 to December 2022, including 16 male and 14 female subjects. None of the subjects had current or previous neurological or orthopedic pathology of the right leg. This study was approved by National Taiwan University Hospital (NTUH) Research Ethics Committee (NTUH IRB approval number: 202209051RINB), and it also was approved by ClinicalTrials.gov (NCT number: NCT05650255); All recruited subjects gave their written informed consent for study participation. 

Table 1 lists detailed information on the subjects. The subjects’ ages ranged between 20 to 32 years old and the ratio of male to female was 1.14, which shows that this study was focused on the young adult stage and was able to reduce the impact of the imbalance between males and females effectively.

### 2.4. The Experimental Protocol for Clinical Trials with IMU Equipment

**Figure 2:**
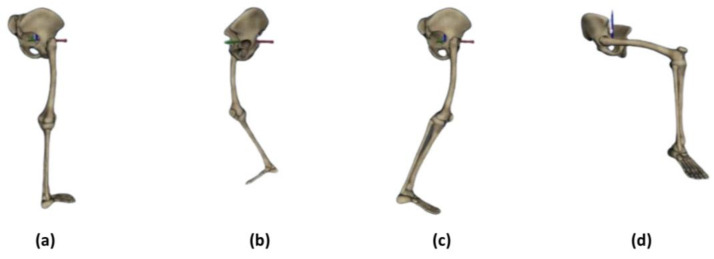
Skeleton pictures of the right foot of the subject were displayed on the iSen 3.0 system when the subject performed different human actions: (**a**) standing, (**b**) walking 01, (**c**) walking 02, (**d**) sitting.

A detailed protocol was designed for this experiment to establish a smooth and accurate measurement of each subject. First, subjects were asked to wear four IMU sensors on their right leg, as shown in Figure 1b. The locations for the four sensors were on the sacrum, right thigh, right calf, and right foot. Second, the IMU sensors and iSen 3.0 analysis program were turned on to ensure successful signal capturing, as shown in Figure 1c. This system allows a selection of sampling rates, such as 25, 100, 200, or 400 Hz. This study used 25 Hz and 100 Hz. Afterward, the subjects were required to stand straight for a while to establish the initial state. Next, subjects were asked to perform a series of “stand–walk–stand–sit” cycles for five to six minutes. “Stand” in this scenario meant that the subject stood straight in his initial state for 15 s, “Walk” meant that the subject walked around the room for 15 to 20 s at their usual pace, and “Sit” meant that the subject sat on a chair for 15 s. After completing the cycle, the data were ready for analysis. The skeleton pictures of the right foot of the subjects were also displayed on the iSen 3.0 system when the subject performed various movements, as shown in Figure 2.

### 2.5. Data Processing

This research developed the necessary deep learning programs using Python to treat and extract the features from the IMU data. The program was written in Python, and PyCharm Community Edition was used as the integrated development environment (IDE). The data processing used an Intel^®^ Core (TM) i7-11700F processor (2.50 GHz) running Win10. As depicted in the previous section, four IMU sensors were placed on the sacrum, right thigh, right calf, and right foot during the experiments to obtain the flexion and extension angles of the subject in his sagittal plane. The iSen 3.0 analysis system translated the collected data above into the joint angles. The system also computed the first and second derivatives to obtain the angular velocity and angular acceleration for the use of more potential features. Also, the validity and repeatability of these parameters from IMU sensors and the iSen 3.0 system were verified using a motion-capture system [46]. These variables comprised the training input signals during the training process.

### 2.6. Posture-Change Detection Algorithm

In the experiment-procedure step, the IMU sensors worn by the subjects provided the main features of the subjects’ sagittal plane movement from the pelvis, the right hip, the right knee, and the right ankle: angular displacement, angular velocity, and angular acceleration, which were the joint parameters relative to the pelvis. Thus, there are a total of 12 data signals in each set. These signals were used as the input for the deep learning network structure for the training process. The network output is then the seven labels of human actions: standing, walking, sitting down, sitting, standing up, starting walking, and stopping walking. Also, for the motivation and application of the generalized deep learning model, the data from Subject 1 with a total duration of 3630 s was chosen to be the training dataset in this study.

**Figure 3:**
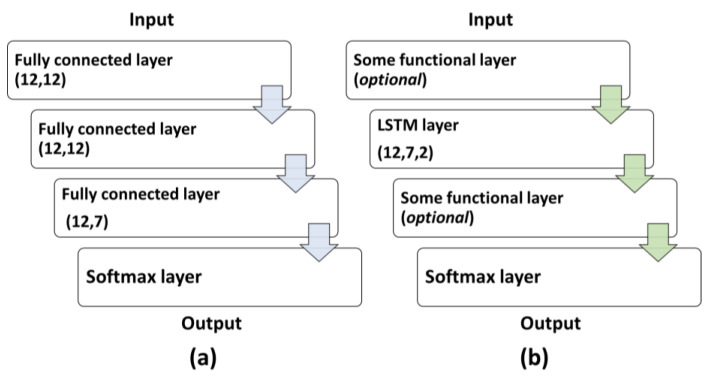
(**a**) The structure of the FNN deep learning system; (**b**) the general structure of the LSTM deep learning system.

This research considered various network components and combinations to identify the most suitable algorithm model for the best intention-identification performance. A critical criterion for the network was that it needed to allow sequential input of the time-domain data so that the system could detect the motion change as the pilot moved around. To achieve this criterion, the authors proposed two choices for network structures: a linear Feedforward Neural Network (FNN) structure and a Long Short-Term Memory (LSTM) network structure. The FNN contained three fully connected layers and a SoftMax layer, as shown in Figure 3a. The network took a matrix with the 2 dimensions of *n* × 12 as an input signal because the data sequence contains three features of the four joints and n was the test time of about 5–6 min. Also, the output of the AI network obtained a matrix with the 2 dimensions of *n* × 7. The fully connected layers were each composed of seven nodes. The Softmax layer also had seven nodes for the seven labels/classifications of human motions. The outcome of the fully connected layers was then processed by a Softmax layer, which rescaled the outputs to fall within the range of [0, 1]. The Softmax function was designed with the constraint that the sum of the output values must equal 1, thereby producing a probability distribution over the possible output labels. Consequently, this criterion ensures that the output corresponds to a single output label with a probability distribution that conforms to the nature of the classification task.

To further examine the possibility of improving performance, this research also tried a 2-layer LSTM structure for comparison (Figure 3b). The reason for adopting the 2-layer LSTM is that it could minimize effort for application on a mobile device with limited computing power. There is also the consideration that the LSTM networks for two layers could decrease the complexity for a regular PC to process.

Various function layers were added before and after the LSTM layer to improve identification accuracy. First of all, the settings for dropout layers reduced the chance of overfitting. The probability of forcing the input to be zeroed (*p* values) could be set to either 0.2 or 0.8. Secondly, the settings for batch-normalization layers could help avoid unbalanced weight growth. Thirdly, adding ReLU layers could help eliminate the negative terms. 

Table 2 lists the ablation study of the LSTM layer groups tested in our design, in which the number in each group stands for the order in which it was placed in the deep learning network. The ratio between the training group and validation group in the training dataset during all network training was 7:3 in this study. In addition to the various LSTM structures, this research also included FNN to compare with the LSTM performances.

### 2.7. Performance Comparison and Transition-Time Analysis

Once the candidate structures had been chosen, the next step was to determine which models would yield better results. In this stage, training loss is a good indicator for gauging the performance of the deep learning structures. The classification functions, such as the sigmoid and Softmax functions or mean square deviation can still impose a high computational load. Alternatively, a relatively straightforward and efficient calculation can be achieved using a cross-entropy classification function. Therefore, this study adopts cross-entropy as the training-loss function. The formula for multiple-sample cross-entropy calculations is as follows:

(1) $$H\left(p,q\right)=-\sum_{i=1}^{m}\sum_{j=1}^{n}p\left(x_{j}\right)\cdot \ln q\left(x_{j}\right)$$

where p and q were the true label distribution and the predicted label distribution, respectively, and *m* and *n* were the sample size and the number of classes. 

**Figure 4:**
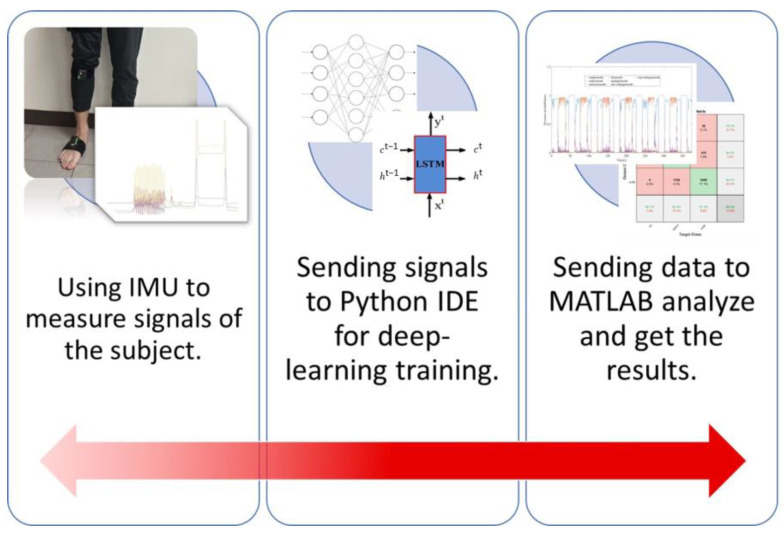
The complete operating flowchart in this study.

Once an effective AI model was selected and trained, it was utilized as the primary algorithm. When input signals for a given case were prepared for the AI model, it generated a series of predictive labels. These outputs were subsequently transmitted to the MATLAB environment (MATLAB 2019b) for post-processing and data analysis to determine the intention-transition time. Figure 4 illustrates the complete operational flowchart of this study, encompassing both the experimental and analytical components.

The MATLAB environment also determined how fast the network could detect human intention change. The performance was defined as the proportion of the time required for the system to identify a change. The accuracy was calculated with the following equation.

(2) $$Accuracy\;for\;intention\;transition\;time\;prediction=1-\frac{\sum {∆T}_{stand/walk/sit}+\sum {∆T}_{start-walk/sit}+\sum {∆T}_{end-walk/sit}}{Total\;experiment\;protocol\;time}\times 100\%$$

Again, ${∆T}_{stand/walk/sit}$, ${∆T}_{start-walk/sit}$, and ${∆T}_{end-walk/sit}$ were the absolute value of the time difference between the true label situation, such as standing, walking, sitting down, sitting, standing up, starting walking, and stopping walking, and the predicted label situation, respectively.

## 3. Results

### 3.1. The IMU Signal Characteristics

**Figure 5:**
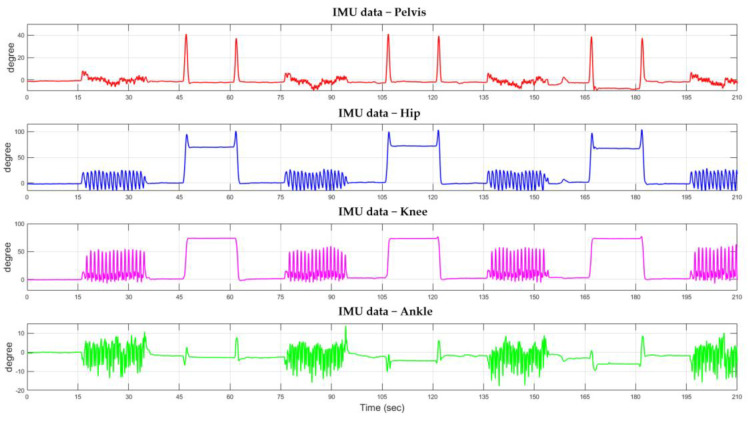
The specific tendency of IMU signals changed when the subject moved.

Figure 5 shows the IMU recorded data signals from the subject’s pelvis, hip on the right side, knee on the right leg, and ankle on the right leg. One can clearly observe specific signal patterns corresponding to different movements of the subject. The IMU signals’ distinctive nature is suitable for human posture-change detection.

In this study, through the IMU sensor and iSen 3.0 software system, the IMU input signal is made up of the angle of joints of the lower limbs, such as the hip, knee, and ankle joints, during motions. Table 3 shows the results of Pearson’s correlation coefficient of the input parameters from IMU sensors with each other, analyzed by SPSS statistic software. The main results indicated that pelvis parameters would be correlated with the parameters of the hip and knee. Also, the parameters of the hip would be correlated with those of the knee, and the parameters of the ankle are only connected with themselves. Meanwhile, it can also be seen obviously from the content of this table that the joints are affected by each other, and the higher the body part is, the more joints it involves. This would also be consistent with general logical thinking.

### 3.2. Data and the Design of Test Posture-Change Sequence

This research used two sets of data from Subject 1, for which sampling was both 25 Hz and 100 Hz, with a total duration of 3630 s to train the proposed deep learning detection networks. 

**Figure 6:**
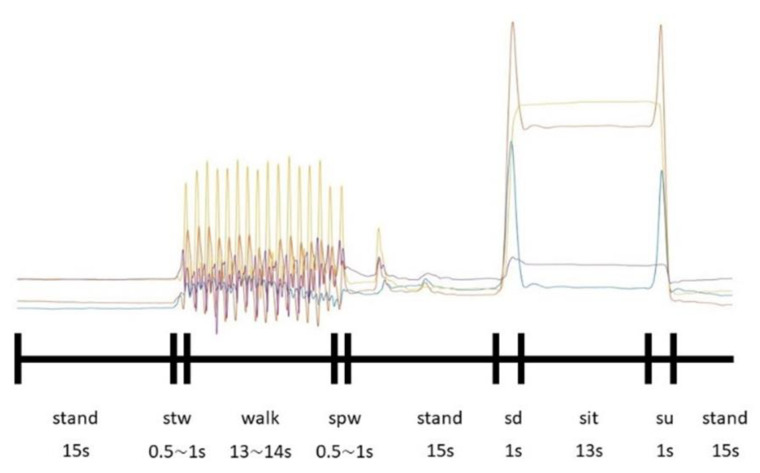
The specific tendency of IMU signals changed when a subject moved. The curves with different colors represented angles with diffident joints.

**Figure 7:**
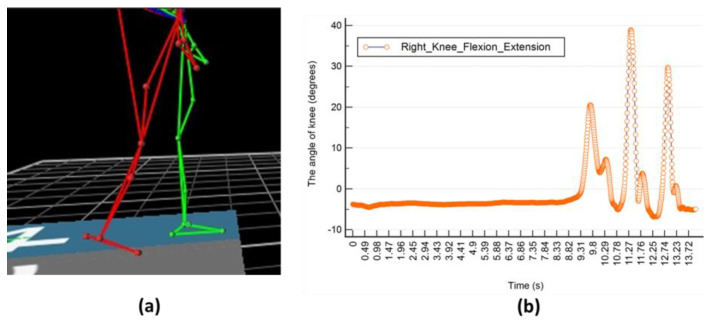
Schematic diagram of kinematic parameters. (**a**) obtained by Vicon device; (**b**) obtained by IMU sensors.

As shown in Figure 6, the signal responses from the different movements were quite distinctive. In the process of labeling, the author used motion-capture technology in the form of a Vicon device to ensure the validity and objectivity of labeling by testing the timestamp from the knee-angle data of IMU sensors, whose sampling rate was 100 Hz, and the Vicon 3D capture system (whose sample rate is 120 Hz) when the subject stood, started to walk, and stopped walking (the timestamp starts from 0 s). When the human body posture started to change, one author himself recorded the frame of Vicon at the moment from which the timestamp can be computed. At the same time, the angle data from IMU sensors was also changed, which one author also recorded and labeled himself. It can be found that the transition time from the two types of device data when posture changes is almost the same and manual labeling for supervised learning was possible, as shown in Table 4 and Figure 7. So, the later experiment would involve three types of motion: standing, walking, and sitting; however, to enable transition detection, we decided to separate the movements into seven events to enable posture-transition detection. The labeling was carried out manually. The seven labels include: standing, walking, sitting down (“sd”), sitting, standing up (“su”), starting walking (“stw”), and stopping walking (“spw”). The sample size for each label above after processing is 157,181, 87,073, 6006, 94,580, 6106, 6055, and 6051, which are considered imbalanced data; however, they can be applied properly, such as by adding appropriate functional layers to the subsequent network architecture. From another perspective, the data of those small sample sizes can serve as outliers, which stand for posture-change time detection, which is the thing that this study focuses on.

### 3.3. The Comparison of Deep Learning Structures between FNN and LSTM Structures

In this section, two primary deep learning structures, FNN and LSTM, were examined. The first dataset comprised data with sampling rates provided by the iSen 3.0 system, with a sampling rate of 25 Hz, and this study included testing on three subjects (Subject 01 to Subject 03). The LSTM structure was selected from Group 5 in Table 2.

**Figure 8:**
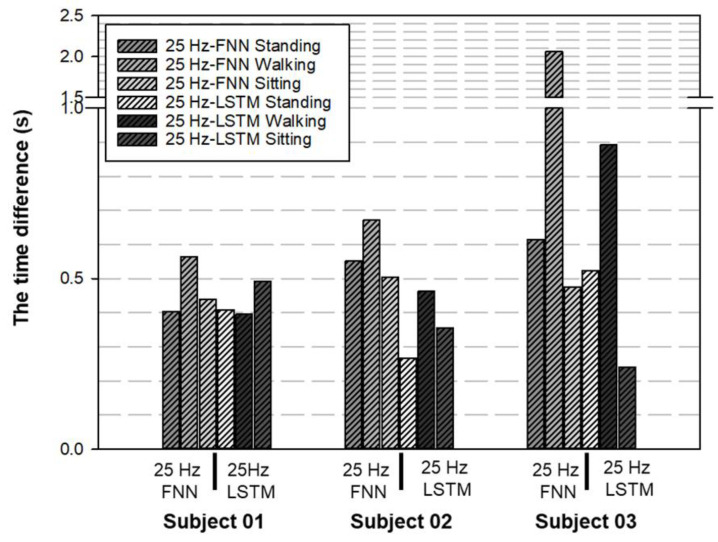
The performance of the time difference ($∆T_{stand}$, $∆T_{walk}$, and $∆T_{sit}$) between FNN and LSTM structure. The stability of the LSTM structure seemed better than that of the FNN for the distributions of the time difference in the case of subject 02 and subject 03.

**Figure 9:**
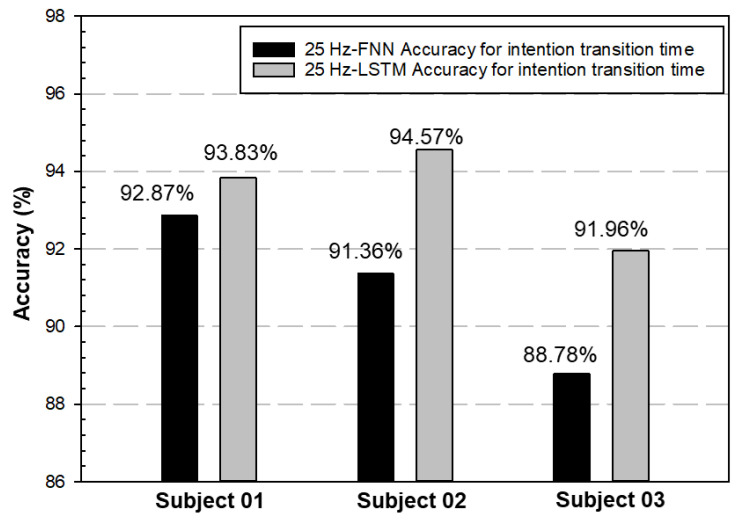
Both methods achieved better results for the measurement of intention-transition time, which ranged from 88% to 94%.

Table 5 shows the computing time of the two deep learning structures. There were no significant differences in the computer time for MATLAB analysis, regardless of whether the structure type was FNN or LSTM (about 3–4 s, accounting for 50% of the total operating time, which included the running time of the AI model for intention-transition-time probability prediction with Python IDE and the running time with MATLAB analysis). However, for the LSTM structure, the training time in Python IDE was 3.6 times that of the FNN structure. The results of the two different structures are shown in Figure 8 and Figure 9, and the accuracy of intention-transition time was compared.

From Figure 9, both methods achieved better results for the accuracy of the intention-transition time, which ranged from 88% to 94%; however, the stability of the LSTM structure was better than the FNN, for the distributions of the identification time. 

**Figure 10:**
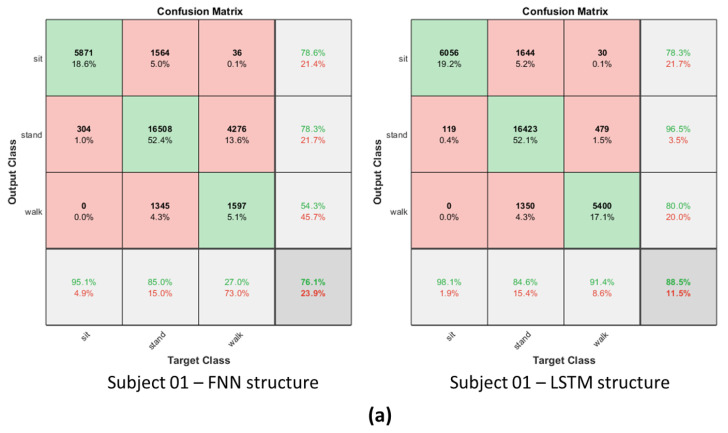

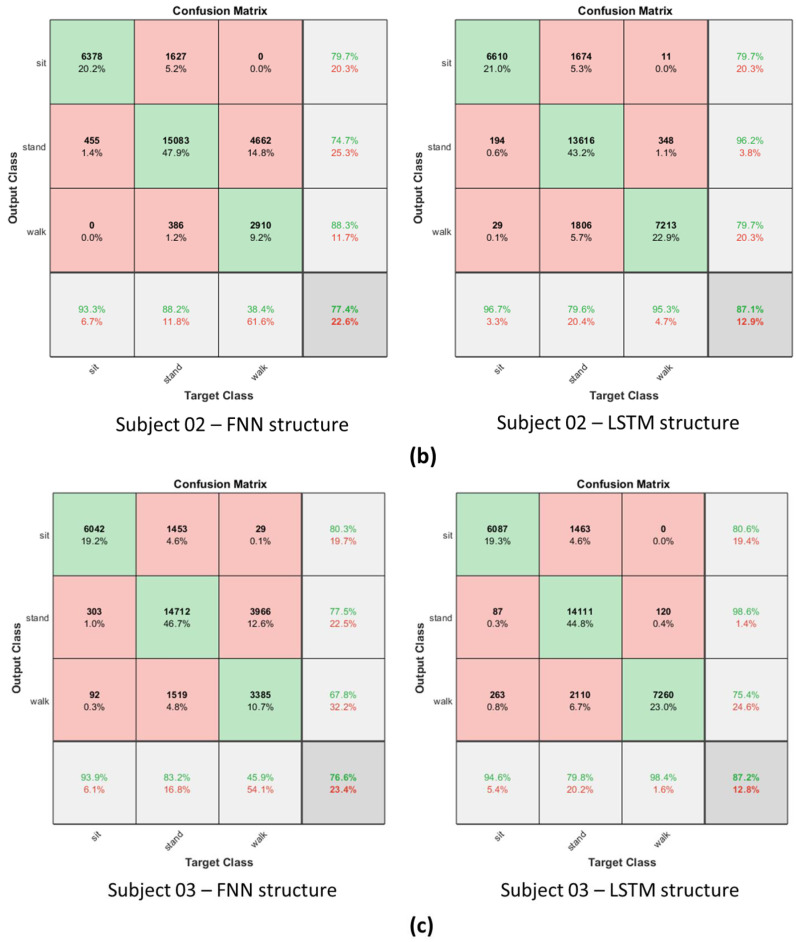
The confusion matrix results in the FNN structure and LSTM structure. (**a**) Subject 01; (**b**) Subject 02; (**c**) Subject 03. (Sampling rate is 100 Hz).

On the other hand, to obtain more precise information, the angle data of IMU sensors with a 100 Hz sample rate are used. In this criterion, the suitable one is still the LSTM structure and its performance is also better for posture-identification accuracy, as shown in Figure 10. As a result, comparing the FNN and LSTM, the latter is faster and more accurate in detecting the motion-switching time. As a result, the LSTM structure with a 100 Hz sampling rate was chosen in this study for later analysis.

### 3.4. Effect of the Sampling Rates

**Figure 11:**
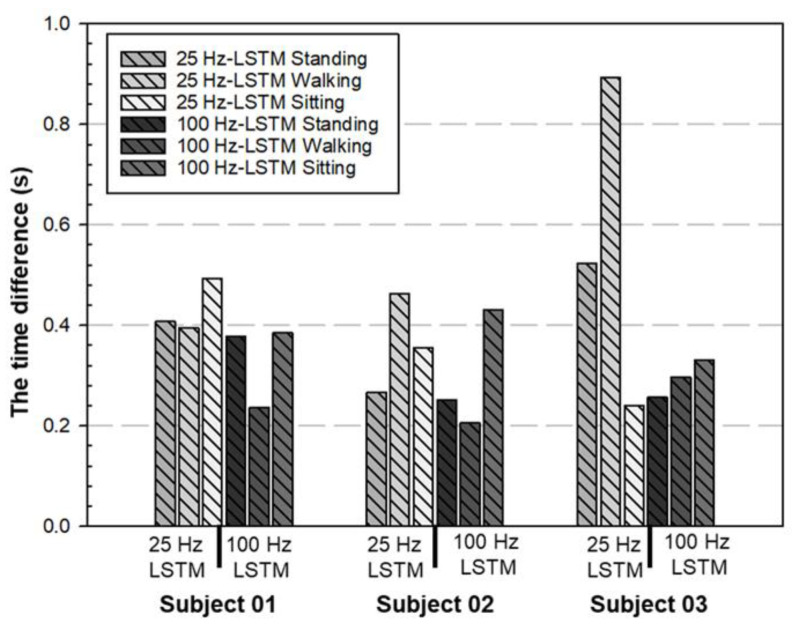
The performance of the time difference ($∆T_{stand}$, $∆T_{walk}$, and $∆T_{sit}$) between different sample rates with LSTM structure. The time difference was decreased when the sample rate was 100 Hz.

**Figure 12:**
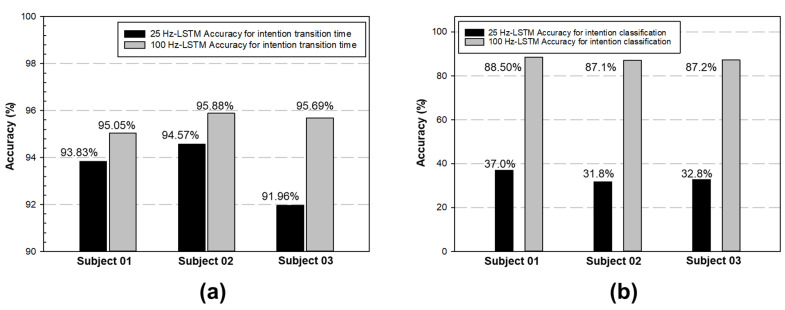
(**a**) The outcome, with both sample rates achieving good results for the measurement of intention-transition time, which range from 91% to 95%.; (**b**) The stability of the LSTM structure with a 100 sample rate seemed better for the accuracy of intention classification.

To examine the effect of the different sampling rates further, the authors also studied the performance of the LSTM network with sampling rates of both 25 and 100. Three subjects (Subject 01 to Subject 03) were chosen for the test again. Figure 11 shows that the performance of the time difference ($∆T_{stand}$, $∆T_{walk}$, and $∆T_{sit}$) with a sampling rate of 100 Hz was better than that of 25 Hz. In other words, the time difference was smaller with a 100 Hz sampling rate. Figure 12 shows that a higher sampling rate also leads to higher accuracy in both the transition detection and motion classification, 91% to 95% and 37% to 88%, respectively. Thus, the selected LSTM structure in Section 3.4 with 100 Hz sampling was confirmed again and chosen for later studies.

### 3.5. The Comparison of Different LSTM Structures’ Performance and Decisions of Deep Learning Models 

After the deep learning type was selected, the next step was to find out which LSTM structure was better for human posture-change detection. As mentioned in Section 2, this study compared five LSTM candidate structures for ablation studies. The loss values at the 150th epoch with the cross-entropy method, the value after validation, and the value at the prediction stage were all recorded to determine which structure achieved the best performance and the outcome of the ablation studies.

**Figure 13:**
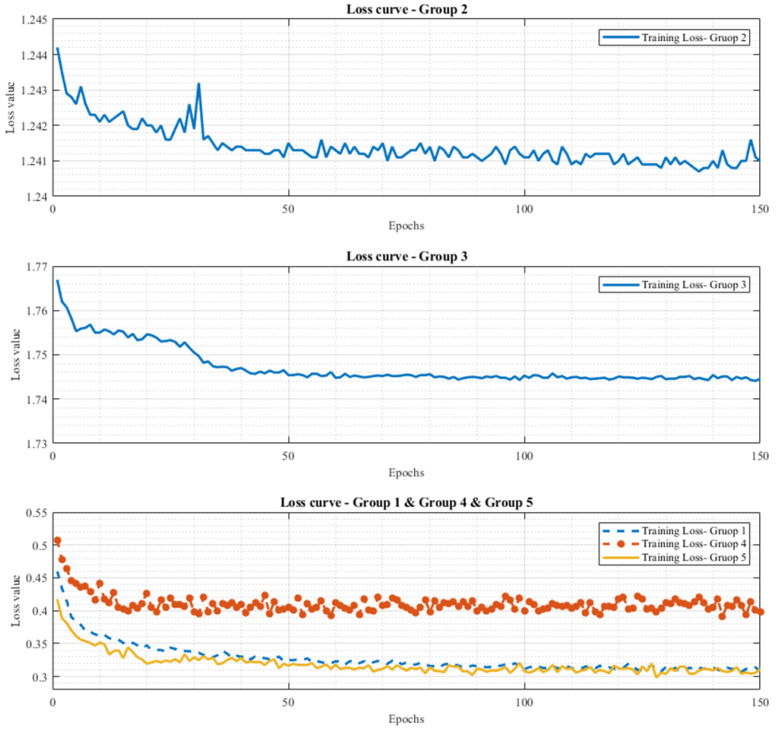
The tendencies of training loss for five LSTM group conditions in the ablation studies.

As shown in Table 6, the loss of Group 5 achieved the minimum training loss, validation loss, and average prediction loss of 0.3112, 0.3141, and 0.3995, respectively. As a result, it was appropriate to choose the network of Group 5 as the identification network. The parameters for the selected structure are also listed in Table 6. Figure 13 shows the tendencies of training loss for the five LSTM groups in the ablation studies. From the outcome of Figure 13, it can be found that if Group 1 was the baseline group and the tendency of the training loss was the performance index for ablation studies, the performance of Group 2, Group 3, and Group 4 was also not better than that of Group 1 and the performance of Group 5 was better than that of Group 1.

### 3.6. The Identification Performance of Human Posture-Transition Time among Different Subjects

Once the main LSTM algorithm was determined, one could test the network’s identification ability on the 30 subjects. Again, the deep learning type was an LSTM network, and the training dataset was from only the data of Subject 1. An additional test was to see whether one could use the same pre-trained network for all the subjects. The sampling rate of all tests was 100 Hz.

**Figure 14:**
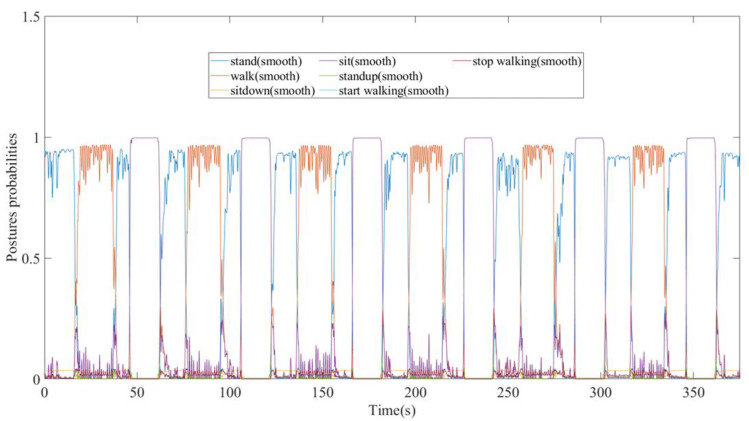
The schematic diagram of the output results from the LSTM model, which represent the probability of each specific intention action vis-à-vis time.

Figure 14 shows the schematic diagram of the test results. The input to the network was the total input variables, as mentioned in Section 2. The output results represented the probability of the identified action (movement) at each time instance. The action with the highest probability value would be considered the recognized human movement at that time. Recall that the primary purpose of this study was to achieve real-time human posture-change identification and to study the possibility of using only the IMU sensor signals to detect human intention when a posture changes. Therefore, this study paid particular focus to the time required for the network to identify the transition. In particular, this study also tried to identify the transition between consecutive postures. 

**Figure 15:**
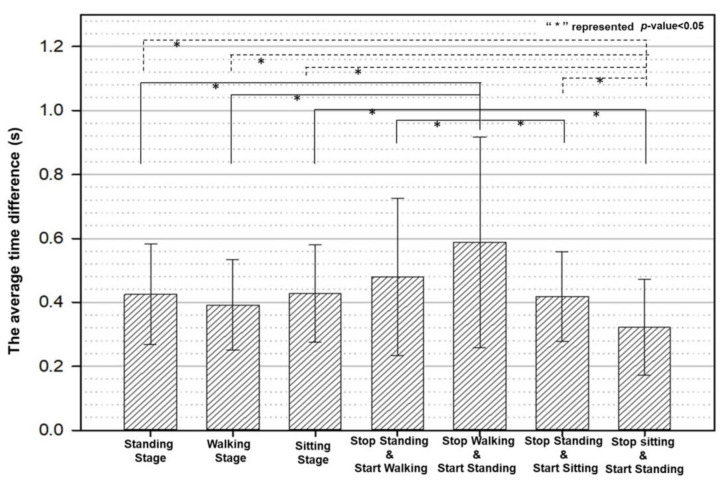
The time difference outcome among 30 different subjects in this study. (Average value ± standard deviation).

Figure 15 shows the statistical analysis of the variation in the identification time. The figure shows the statistics of the thirty subjects on the identification-time differences of the seven postures: “standing”, “walking”, “sitting”, “stop standing and start walking”, “stop standing and starting sitting”, and “stop sitting and start standing” (${∆T}_{stand/walk/sit}$, ${∆T}_{start-walk/sit}$ and ${∆T}_{end-sit}$). One notices that the identification time for the postures was all less than 0.5 s (0.4255 s, 0.39 s, 0.429 s, 0.479 s, 0.405 s, and 0.3188 s, respectively). The figure also shows a significant difference in the statistics under the t-test if the analysis of the posture was based on “stop walking and start standing”. One also observes significant differences in the t-test result for all postures when the comparison was based on “stop sitting and start standing” except for “stop standing/walking and start walking/standing”. The result indicated that the transition identification of “stop sitting and start standing” was close to the ground truth. The detailed results are listed in Table 7.

In addition to the transition-time values, Table 7 also shows the identification accuracy. The definition of identification accuracy is in Section 2. This research also utilized the confusion matrix for all drivers (participants) to judge the identification accuracy. From the accuracy results in Table 7, the network achieved 94.44% accuracy for transition time and 85.87% accuracy for posture-change detection.

## 4. Discussion

Instead of just identifying the posture, this research focuses on how fast a network can detect a posture transition, a function essential to human compliance robotics. The main goal is to catch the instant when humans intend to change gait or posture using IMU sensor signals. In contrast to similar studies, this research strongly emphasizes pinpointing the moment when a person’s intention shifts, with the aim of facilitating the adequate switching of control laws in exoskeletons. What is more, these pieces of information on human intention cannot be obtained directly from the exoskeleton motor.

This study identified standing, walking, and sitting as the three main scenarios for exoskeleton initial activities. We also stress the importance of accurately detecting the instant when a human initializes a posture change. Initial observation of the experimental results reveals that the changes in the flexion and extension angles were not immediate when the subjects switched between standing and walking. The response of the general sagittal plane flexion and extension angle (Figure 6) allows for an easy distinction between “sd”—sitting down—and “su”—standing up. Here, “sd” means that the subject changed posture from standing to sitting, while “su” means the subject stood up from a sitting posture. Both movements take about one second and show quite distinctive characteristics compared to “sitting still.” In particular, this research separated the transition phase as separate postures: “stw”—starting walking—and “spw”—stopping walking—for precise identification of the moment when posture changes. Here, “stw” means the subject changed his posture from standing to walking, while “spw” means the subject slowed down from the walking state. Both movements took about half a second to one second for all the subjects. For the same reason, we have added the labels “sitting down”, “standing up”, “start walking”, and “stop walking”. It is worth pointing out that these movements also correspond to very distinctive joint-torque characteristics.

This research compared FNN and LSTM as candidate networks and defined the difference between the actual posture-switching time and the identified switching time as the detection time. FNN required about 0.6 to 1.9 s to detect a transition into walking, while LSTM required about 0.4 to 0.8 s, as shown in Figure 8, which used the same case data with a 25 Hz sampling rate. As a result, LSTM could detect the transition moment much faster. The fastest it could detect a transition is 0.24 to 0.49 s in detecting a sitting down movement. Also, LSTM was a recurrent neural network (RNN) suitable for exoskeleton controller applications. 

There was a substantial improvement in the detection accuracy when the sampling rate of the angle information of the IMU sensors increased from 25 to 100 Hz (Figure 12). As the dataset from different test subjects increased, we saw that the network trained for one subject could detect posture changes in all subjects equally fast, whether they be male or female. The average detection time was 0.4 s with a *p*-value of 0.05 (Figure 15 and Table 7). Although the response time was not instantaneous, it was fast enough for practical purposes. The test result showed that the system proposed in this study could effectively reflect the pilots’ initial intention to change posture and that training a preliminary controller network that already performed well for general use was possible.

Compared to previous research in this field, it has been demonstrated that linear discriminant analysis (LDA) and random forest (RF) can effectively predict a subject’s intention to initiate movement. Notably, the average prediction transition time was approximately 31 × 10^−4^ seconds and 11 × 10^−5^ seconds, respectively [31,33]. It is important to note that these studies predominantly focused on the upper limbs and arms only, characterized by relatively simple joint freedom and kinematic parameters.

On the other hand, a separate study indicated that the prediction times for transitions varied from 277 ms to 488 ms when considering walking as the reference state [43]. However, it is worth mentioning that achieving such results necessitated the integration of various sensors, including IMU, EMG, and other types of sensors. In contrast, this study achieved an average prediction time of approximately 400 ms for lower limb action transitions. Remarkably, this was accomplished using just one type of sensor (IMU) in combination with an effective LSTM algorithm. This outcome indicates that comparable results to previous research can be obtained with more streamlined sensor configurations.

Furthermore, based on the results of ablation studies involving various LSTM deep learning structures, it was observed that placing the Dropout layer before the LSTM layer with a *p* value of 0.4 did not outperform Group 1 (in the case of Group 3). Additionally, when the Batch Normalization layer and Dropout layer were situated before or after the LSTM layer, their performance still did not surpass that of Group 1 (in the case of Group 2) if we consider Group 1 as the baseline group and the trend of training loss as the performance metric for ablation studies. It becomes evident that the primary functions of the Batch Normalization layer and Dropout layer, which include regularization, overfitting prevention, and managing weight imbalances in the network architecture, apply to situations involving imbalanced data. However, the sequence in which these layers are added can significantly influence the final performance index and target-prediction performance. This information would be very useful for the modification of the LSTM network architecture.

To sum up, this study built up a deep learning algorithm and structure using only IMU sensors that started with some initial posture or intention prediction, which proved that AI technology could be further applied to the more complicated field of human posture change or exoskeleton control by human-intention detection.

## 5. Limitations

The proposed method showed a good outcome in measuring or detecting human intention in this study, but it could be worth further exploring. First, inertial measurement units (IMUs) were used for the current study. Other wearable sensors could be combined with this, such as electromyography (EMG), torque sensors, or the application of sensor-fusion technology with a more modern AI algorithm [47], and the relationship between the unitary duration of activities and the duration of all activity switches for kinematic modeling in the HAR research field [48] might be included in the next phase of research to obtain more meaningful data. Second, the present research only considered the sagittal plane joint angles of the subjects’ right feet. It would be interesting also to consider the influence of the angles in the transverse and the coronal plane. Third, the postures studied were relatively simple; different postures or gaits, actual implementation problems, and combination with an exoskeleton could be considered in future studies to match real-life scenarios and engineering applications.

## 6. Conclusions

This research proposed two machine learning network structures, a linear Feedforward Neural Network (FNN) and a Long Short-Term Memory (LSTM), to detect the switching of human postures, and three everyday initial human postures were used, including standing, walking, and sitting. The system used only IMU sensors to enable portability. To speed up posture-change detection, the authors introduced the transition stages as distinct features for identification. Comparing the detection results showed that the LSTM model with a 100 Hz sampling rate achieved the best results for posture-change detection time and posture-classification accuracy, about 94.44% and 85.87%, respectively. Even though LSTM took longer to train, it does not affect the implementation requirement.

Further investigation of the sampling-rate effect showed that a faster sampling rate (four times faster) improved the accuracy of posture-change detection from an average of about 91% to 95%. In addition to the network design, this research also showed that using a network trained for one subject was adequate for use on the other subjects. This result suggests the feasibility of selecting personalized or generalized deep learning models in related applications for the field of detecting human posture changes.

## Figures and Tables

**Table 1:** The information on the 30 healthy voluntary subjects.

	**Parameters**
Number of subject’s sex	Male: 16; Female: 14
The ratio of male to female	1.14
	**Mean ± SD**
The mean of subjects’ age (years)	26 ± 5.92 (Range: 22–50)
The mean of subjects’ height (cm)	165.57 ± 8.94 (Range: 142–179)
The mean of subjects’ weight (kg)	61.07 ± 12.34 (Range: 39–95)
Lengths of right thigh of subjects (cm)	41.43 ± 5.43
Lengths of right calf of subjects (cm)	40.37 ± 4.35
Lengths of right foot of subjects (cm)	24.53 ± 1.94

**Table 2:** The ablation study of the LSTM layer groups in this study.

	Group 1	Group 2	Group 3	Group 4	Group 5
Dropout layer (*p* = 0.2)	1				
Dropout layer (*p* = 0.4)		3	1		
Dropout layer (*p* = 0.5)			5		
Dropout layer (*p* = 0.8)	6			2	4
LSTM layer	2	2	2	1	1
Fully connect layer 1	3		3		2
Fully connect layer 2	7	4	6	3	5
Batch Normalization layer	4	1			3
Leakly ReLu layer	5		4	4	6
Softmax layer	8	5	7	5	7

**Table 3:** The correlation coefficient table of the input parameters from IMU sensors. An orange background block represents that the correlation coefficient result is greater than ±0.3.

	Pelvis Angle	Pelvis Angular Velocity	Pelvis Angular Acceleration	Hip Angle	Hip Angular Velocity	Hip Angular Acceleration	Knee Angle	Knee Angular Velocity	Knee Angular Acceleration	Ankle Angle	Ankle Angular Velocity	Ankle Angular Acceleration
**Pelvis** **Angle**	1	0.01136	−0.24293	**0.578343**	−0.01739	−0.14509	**0.391564**	−0.0797	−0.00427	0.176395	0.016812	−0.01739
**Pelvis** **Angular** **Velocity**		1	−0.04938	0.002561	**0.549951**	−0.12353	0.080105	0.074217	**−0.4339**	−0.07015	0.118572	0.091082
**Pelvis** **Angular** **Acceleration**			1	−0.08809	0.054116	**0.646527**	−0.03164	**0.517422**	0.132756	−0.08723	−0.18378	0.054194
**Hip** **Angle**				1	−0.00791	−0.17426	**0.922779**	−0.13341	−0.02437	**0.421537**	0.029337	−0.00376
**Hip** **Angular** **Velocity**					1	−0.04477	0.205918	0.249638	**−0.50045**	−0.16232	0.019327	0.137285
**Hip** **Angular** **Acceleration**						1	−0.08519	**0.629106**	**0.408148**	0.005521	−0.28019	−0.04438
**Knee** **Angle**							1	−0.01249	−0.2038	**0.410049**	0.037825	0.022378
**Knee** **Angular** **Velocity**								1	−0.06072	−0.11249	−0.20555	0.194528
**Knee** **Angular** **Acceleration**									1	0.167935	−0.26501	−0.19381
**Ankle** **Angle**										1	−0.04289	**−0.44771**
**Ankle** **Angular** **Velocity**											1	−0.0941
**Ankle** **Angular** **Acceleration**												1

**Table 4:** The time label from the Vicon device and IMU sensors (the timestamp of start to walk and stop to walk). The experiment was performed in triplicate for the same subject.

	The Time Label from IMU Sensors (Unit: s) (Mean ± S.D.)	The Time Label from the Vicon Device (Unit: s) (Mean ± S.D.)
Start to walk	7.4150 ± 0.473	7.65826 ± 0.709
Stop to walk	11.9350 ± 0.544	11.16389 ± 0.359

**Table 5:** The basic time information with two deep learning structures.

Training Dataset	Sample Rate (Unit: Hz)			Experiment Time (Unit: s)		
	25			3630		
	Deep Learning Type	Sample Rate (Unit: Hz)	Experiment Time (Unit: s)	Training Time in Python IDE (Unit: mins)	MATLAB Analysis Time (Unit: s)	
Subject 01	FNN	25	375	24.60	3.804	About 50% of the total operating time, which included the running time of the AI model with Python IDE and the running time with MATLAB analysis.
Subject 02			315		3.888	
Subject 03			315		3.789	
Subject 01	LSTM	25	375	88.80	3.731	
Subject 02			315		3.856	
Subject 03			315		3.905	

**Table 6:** The performance comparisons between different LSTM group conditions.

Parameters	Learning Rate: 0.001	Weight Decay: 0.00001		Loss Function: Cross-Entropy
	Epochs: 150	Batch Size: 10		Use One GPU Device
Training Dataset	The Number of Features: 12		The Number of Labels: 7	
	The Number of Samples: 217,880 Samples from One Person. (3630 s, Whose Sample Rate Was 100 Hz)			
Group Types	Training Stage		Prediction Stage	Training Time in Python IDE (mins)
	Training Loss	Validation Loss	Average Prediction Loss	
Group 1	0.3082	0.3101	0.4109	206
Group 2	1.2410	1.2409	0.6357	229
Group 3	1.7446	1.9525	0.4520	241
Group 4	0.3980	0.3982	0.4362	244
Group 5	0.3112	0.3141	0.3995	249

**Table 7:** The statistical outcome information from the LSTM model between 30 different drivers in this study.

Software Operating Time	Mean ± SD
Python algorithm with LSTM model for intention-transition-time probability prediction (s)	6.76667 ± 1.30472 (80.37% of operating time)
Analysis with MATLAB for intention-transition-time prediction (s)	1.65217 ± 0.71406 (19.63% of operating time)
**Statistic results of the time difference when human posture changed**	**Mean ± SD**
In the standing stage (s)	0.42557 ± 0.16049
In the walking stage (s)	0.39019 ± 0.14458
In the sitting stage (s)	0.42941 ± 0.15568
Stop standing and start walking (s)	0.47986 ± 0.25106
Stop walking and start standing (s)	0.59732 ± 0.33405
Stop standing and start sitting (s)	0.40483 ± 0.12591
Stop sitting and start standing (s)	0.31880 ± 0.15251
**Accuracy for posture-change detection**	**Mean ± SD**
Accuracy for prediction of intention classification (%)	85.87 ± 7.49
Accuracy for prediction of intention-transition time (%)	94.44 ± 1.70

## Data Availability

Data are available on request due to ethical restrictions. The data presented in this study are available on request from the corresponding author. The data are not publicly available due to regulations of the Research Ethics Committee.
